# General treatment principles for fracture-related infection: recommendations from an international expert group

**DOI:** 10.1007/s00402-019-03287-4

**Published:** 2019-10-29

**Authors:** Willem-Jan Metsemakers, Mario Morgenstern, Eric Senneville, Olivier Borens, Geertje A. M. Govaert, Jolien Onsea, Melissa Depypere, R. Geoff Richards, Andrej Trampuz, Michael H. J. Verhofstad, Stephen L. Kates, Michael Raschke, Martin A. McNally, William T. Obremskey, On behalf of the Fracture-Related Infection (FRI) group, Willem-Jan Metsemakers, William T. Obremskey, Martin A. McNally, Nick Athanasou, Bridget L. Atkins, Olivier Borens, Melissa Depypere, Henrik Eckardt, Kenneth A. Egol, William Foster, Austin T. Fragomen, Geertje A. M. Govaert, Sven Hungerer, Stephen L. Kates, Richard Kuehl, Leonard Marais, Ian Mcfadyen, Mario Morgenstern, T. Fintan Moriarty, Peter Ochsner, Alex Ramsden, Michael Raschke, R. Geoff Richards, Carlos Sancineto, Charalampos Zalavras, Eric Senneville, Andrej Trampuz, Michael H. J. Verhofstad, Werner Zimmerli

**Affiliations:** 1 Department of Trauma Surgery, University Hospitals Leuven, Leuven, Belgium; 2 Department of Orthopaedic and Trauma Surgery, University Hospital Basel, Basel, Switzerland; 3 Department of Infectious Diseases, Gustave Dron Hospital, University of Lille, Lille, France; 4 Orthopedic Department of Septic Surgery, Orthopaedic-Trauma Unit, Department for the Musculoskeletal System, CHUV, Lausanne, Switzerland; 5 Department of Trauma Surgery, University of Utrecht, University Medical Center Utrecht, Utrecht, The Netherlands; 6 Department of Laboratory Medicine, University Hospitals Leuven, Leuven, Belgium; 7 AO Research Institute Davos, Davos, Switzerland; 8 Center for Musculoskeletal Surgery, Berlin Institute of Health, Charité-Universitätsmedizin Berlin Corporate Member of Freie Universität Berlin, Humboldt-Universität zu Berlin, Berlin, Germany; 9 Department of Trauma Surgery, Erasmus University Medical Centre, Rotterdam, The Netherlands; 10 Department of Orthopaedic Surgery, Virginia Commonwealth University, Richmond, USA; 11 Department of Trauma Surgery, University Hospital of Münster, Münster, Germany; 12 The Bone Infection Unit, Nuffield Orthopaedic Centre, Oxford University Hospitals, Oxford, UK; 13 Department of Orthopaedic Surgery and Rehabilitation, Vanderbilt University Medical Center, Nashville, TN USA

**Keywords:** Fracture-related infection, Treatment, Diagnosis, Outcome, Fracture, Infection

## Abstract

Fracture-related infection (FRI) remains a challenging complication that creates a heavy burden for orthopaedic trauma patients, their families and treating physicians, as well as for healthcare systems. Standardization of the diagnosis of FRI has been poor, which made the undertaking and comparison of studies difficult. Recently, a consensus definition based on diagnostic criteria for FRI was published. As a well-established diagnosis is the first step in the treatment process of FRI, such a definition should not only improve the quality of published reports but also daily clinical practice. The FRI consensus group recently developed guidelines to standardize treatment pathways and outcome measures. At the center of these recommendations was the implementation of a multidisciplinary team (MDT) approach. If such a team is not available, it is recommended to refer complex cases to specialized centers where a MDT is available and physicians are experienced with the treatment of FRI. This should lead to appropriate use of antimicrobials and standardization of surgical strategies. Furthermore, an MDT could play an important role in host optimization. Overall two main surgical concepts are considered, based on the fact that fracture fixation devices primarily target fracture consolidation and can be removed after healing, in contrast to periprosthetic joint infection were the implant is permanent. The first concept consists of implant retention and the second consists of implant removal (healed fracture) or implant exchange (unhealed fracture). In both cases, deep tissue sampling for microbiological examination is mandatory. Key aspects of the surgical management of FRI are a thorough debridement, irrigation with normal saline, fracture stability, dead space management and adequate soft tissue coverage. The use of local antimicrobials needs to be strongly considered. In case of FRI, empiric broad-spectrum antibiotic therapy should be started after tissue sampling. Thereafter, this needs to be adapted according to culture results as soon as possible. Finally, a minimum follow-up of 12 months after cessation of therapy is recommended. Standardized patient outcome measures purely focusing on FRI are currently not available but the patient-reported outcomes measurement information system (PROMIS) seems to be the preferred tool to assess the patients’ short and long-term outcome. This review summarizes the current general principles which should be considered during the whole treatment process of patients with FRI based on recommendations from the FRI Consensus Group.

**Level of evidence:** Level V.

## Introduction

Fracture-related infection (FRI) remains a major complication that can result in permanent functional loss or even amputation in otherwise healthy patients. Infection prevention is of utmost importance to improve patient outcome [1]. Despite prevention measures, FRI still occurs and causes significant morbidity in 1–30% of all orthopaedic trauma patients [1]. For these reasons, standardization of diagnosis and treatment is critical to improve outcome. Most treatment principles are currently based on research that has been performed on prosthetic joint infection (PJI). However, FRIs have unique features (i.e. fracture, bone healing, soft tissue injury) that need to be considered [2]. A first step towards standardization of the diagnosis was achieved by the international consensus definition on FRI, which has recently been published [3]. As a next step, treatment principles for FRI and assessment of outcome should also become internationally standardized.

**Fig. 1:**
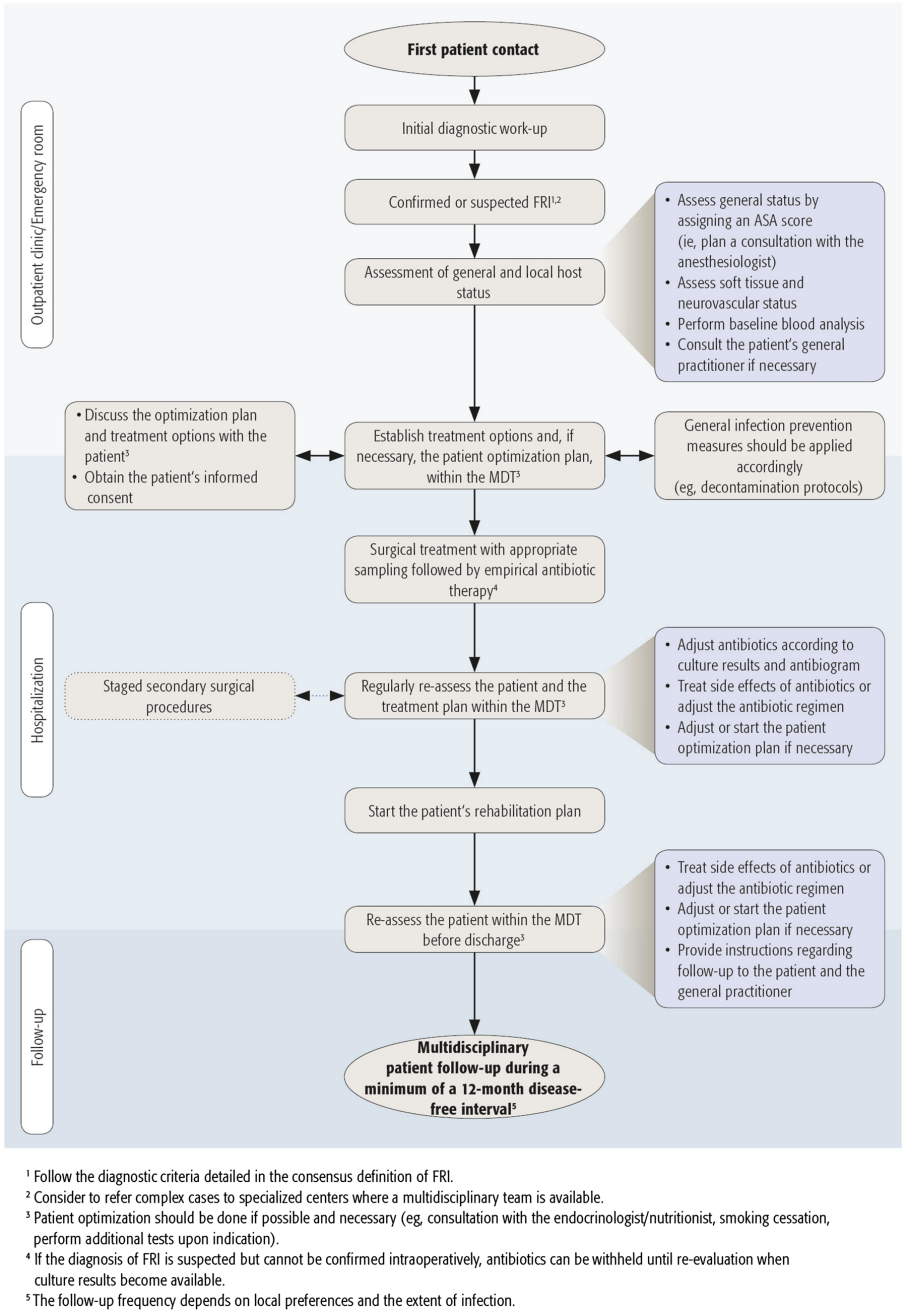
Flowchart representing the optimal treatment course for a patient with FRI

This review provides a summary of the general principles with respect to the treatment of the patient suffering from FRI. We also present a step-wise approach to allow clinicians to address this often difficult clinical problem (Fig. 1).

## General principles

### Diagnosis of FRI

A first step in the work-up of the FRI patient is a well-established diagnosis. Recently, an internationally accepted definition, including diagnostic criteria, was developed for FRI [3]. These diagnostic criteria were updated in a more recent publication [4]. Two levels of certainty around diagnostic features were defined. Criteria for infection can be confirmatory (infection is definitely present) or suggestive. The presence of confirmatory signs should prompt the initiation of a treatment course for FRI, as displayed in Fig. 1. Suggestive signs should motivate the medical team to further investigate the possibility of the presence of an FRI [3, 4]. Confirmatory and suggestive criteria for the diagnosis of FRI are listed in Table 1.

**Table 1:** Diagnostic criteria for FRI [3, 4]

Confirmatory criteria	Suggestive criteria
Clinical signs Fistula Sinus Wound breakdown Purulent drainage or the presence of pus	Clinical signs Local/systemic (e.g. local redness, swelling, fever) New-onset joint effusion Persistent, increasing or new-onset wound drainage
Microbiology Phenotypically indistinguishable pathogens identified by culture from at least 2 separate deep tissue/implant specimens	Laboratory signs Increased serum inflammatory markers (ESR, WBC, CRP)
Histopathology Presence of microorganisms in deep tissue specimens, confirmed by using specific staining techniques for bacteria and fungi Presence of > 5 PMNs/HPF in chronic/late-onset cases (e.g. fracture nonunion) [5]	Radiological and/or nuclear imaging signs microbiology Pathogenic microorganism identified from a single deep tissue/implant specimen

*ESR* erythrocyte sedimentation rate, *WBC* white blood cell count, *CRP* C-reactive protein, *PMNs* polymorphonuclear neutrophils, *HPF* high-power field

A detailed overview of the available diagnostic criteria is presented in affiliated articles of the FRI consensus group [3, 4].

### Classification of FRI

Fracture-related infection can be classified according to the time to onset of symptoms after fracture fixation, dynamics of symptoms, route of infection, location, fracture stability and union status, host type, soft-tissue envelope and disease-causing pathogens [6].

Time of onset of symptoms after fracture fixation is the most commonly used and applied modality to classify FRI [2]. Willenegger and Roth [7] classified FRIs as early (< 2 weeks), delayed (3–10 weeks) and late (> 10 weeks). This classification is widely adopted since it represents the time-dependent pathophysiologic changes of FRIs and may affect treatment decisions [2]. Infection presenting early after osteosynthesis is mainly caused by highly virulent pathogens (e.g. *Staphylococcus aureus*). The diagnosis is often made by clinical assessment, since these patients often present with classic local signs of infection as well as wound drainage, which may be accompanied by a local or systemic inflammatory reaction. In this stage, bacteria can attach to surfaces and form a biofilm, however, this “early” biofilm may still be in an ‘immature’ phase [8]. Delayed presentation represents a grey zone with a maturing biofilm and possible bone invasion of the pathogens. The delay may be due to less virulent organisms or to inadequate early antibiotic use, partially suppressing an early onset of infective symptoms. In late infections, a mature biofilm as well as bone necrosis and osteolysis may be seen, necessitating thorough debridement and implant removal/exchange [2, 9]. The causative pathogens can be very diverse. This has been confirmed by various preclinical and clinical studies showing poor results of implant retention in late FRIs [2].

Other authors differentiated acute and chronic infections with a cut-off of 6 weeks after fracture fixation [10, 11]. There is no robust evidence for a clear time-dependent classification of FRI. Infection develops gradually and treatment decisions may be dictated more by the features present at presentation.

Several important factors, other than time after fracture fixation, influence the decision-making process with respect to the main surgical concepts of FRI treatment: debridement and retention versus debridement and removal or exchange. Recent publications have presented more elaborate classifications like the “Seven-Item Comprehensive Classification System” [12] and the BACH [13] classification system. However, currently no classification is available that is tailored specifically to the FRI patient, is easy to use in daily practice and considers essential factors to guide treatment.

In the near future a consensus should be reached on which factors need to be integrated in a new FRI classification. The main purpose of such a classification should be to guide antimicrobial and surgical treatment decisions. It would also aid the identification of complex cases, where a multidisciplinary approach in a specialized center should be strongly considered [13].

### Multidisciplinary approach

There is increasing evidence that multidisciplinary teamwork and collaboration between healthcare workers are essential to improve patient outcome [14, 15]. The use of antibiotic stewardship programs is already a well-known example of such a multidisciplinary approach for the treatment of infections. They are defined as coordinated interventions designed to improve and measure the appropriate use of (antibiotic) agents by promoting selection of the optimal (antibiotic) drug regimen, including dosing, duration of therapy and route of administration [16]. This improves patient safety, outcome, and, combined with reduced readmission rates, reduces healthcare costs without compromising quality of care [17–20]. According to guidelines from the Infectious Diseases Society of America (IDSA), infectious disease (ID) physicians and clinical pharmacists should be the core members of the antibiotic stewardship program, but microbiologists and the input from administrative and information personnel can also be of great importance [21]. On the other hand, as recently stated by Pulcini et al. [22], the composition of these teams is flexible and should be based on existing international recommendations and adapted to local resources and expertise. Recently, a multidisciplinary approach, consisting of collaboration between orthopaedic trauma surgeons, the hospital’s infection control department, nurses and anesthesiologists as primary team members, has been described to guide FRI prevention strategies [1]. Consistent with this and the concept of antibiotic stewardship programs, a multidisciplinary approach for the treatment of FRI patients should be adopted, in which surgeons are key members of the team, since surgical management plays a critical role. However, studies within this field are scarce [11, 23, 24].

With respect to the treatment of FRI, the essential members of the multidisciplinary team (MDT) require expertise in bone and soft tissue reconstruction, microbiology, antibiotic treatment and advanced imaging. Specialists with a background of the following disciplines can therefore be involved in the MDT for the treatment of FRI: orthopaedic trauma surgeons, ID specialists or internists, microbiologists, plastic and reconstructive surgeons, clinical pharmacists, musculoskeletal radiologists, nuclear medicine specialists, anesthesiologists, physical medicine and rehabilitation physicians, physiotherapists, endocrinologists/nutritionists and (specialist) nurses. Of course, not all members should be involved in every case, but a minimum of three disciplines including surgeons, ID specialists or internists and clinical pharmacists, seems advisable. The exact composition of the MDT will ultimately depend on patient needs, local resources and preferences. A pre-operative assessment should precede the definitive treatment plan (Fig. 1).

Due to the high level of complexity that is often associated with FRI, and the necessity to involve multiple specialties in treatment decision making, it is recommended that physicians strongly consider the referral of complex cases to more specialized centers (centralization) where such an MDT is available.

### Assessment of the patients’ general and local health status

Factors compromising the patient’s general health status increase the risk of postoperative complications. Therefore, the nature of treatment should be customized according to the patient’s general health and condition of the limb. Again, scientific evidence on this topic for FRI is scarce.

The host classification system that is often referred to was developed by Cierny et al. [25] and helps to determine treatment options for osteomyelitis. This classification combines anatomic, clinical and radiologic features. The host is characterized as either class A, B or C. No systemic or local compromising factors are present in host class A, while patients in host class C are so severely compromised that surgical treatment would imply a far greater risk than the osteomyelitis in itself [25]. Bowen et al. applied the host classification described by Cierny et al. in combination with the Gustilo-Anderson classification for open fractures. They found that host and Gustilo-Anderson classification as well as fracture location and tobacco use are predictors of infection in open fractures [26]. The classification by Cierny et al. was also adapted to guide treatment for patients with infection after total hip arthroplasty [27]. The latter classification divides patients into three categories based on infection type (I, II or III), systemic host grade (A, B or C) and local wound and extremity grade (1, 2 or 3). Host status is a predictor of treatment outcome: a higher host grade is associated with increased treatment failure [25–27]. However, this classification [27] is difficult to extrapolate to patients with FRI, because it includes a time component that is arbitrary and a local extremity status that specifically refers to prosthetic joints.

The American Society of Anesthesiologists physical classification (ASA score) aims to determine host general health prior to surgery and classifies patients into five categories of general medical illness, independent of the planned surgical procedure [28]. Although it is a subjective tool, it is correlated with more objective comorbidity indices such as the Charlson comorbidity index and the revised cardiac risk index (RCRI) and has (moderate) predictive value regarding perioperative risk assessment, perioperative mortality and complication rates as well as postoperative outcomes [28–31]. The inter-rater reliability and predictive value of this score increase when the cases correlate to the anesthesiologist’s specialty [32]. Because it is a simple, easy-to-use scale that is already well known and frequently used to assess orthopaedic trauma patients [28–32], we recommend its implementation in the risk stratification of FRI patients.

In addition, evaluation of the patient’s limb status is of critical importance. As the clinical presentation of orthopaedic trauma patients is often extremely variable—ranging from open fractures to chronic/late onset cases—not only the soft tissue and neurovascular status should be assessed by the treating surgeon, but also the overall functionality of the affected limb.

### Host optimization

Depending on the assessment of the general and local health status, the patient should, if possible, be optimized. Comorbidities like poor nutritional status, obesity, age-related diseases and all additional factors that influence wound healing can adversely affect treatment outcome [33]. In addition, regardless of the type of surgery, compliance with general infection prevention measures (e.g. preoperative washing and decontamination, appropriate surgical skin antisepsis, etc.) remains an important factor (Fig. 1) [1].

Regarding host optimization, it is known that smoking delays wound healing, and therefore the patient should be encouraged to cease smoking as soon as possible prior to surgery and at least for the duration of the healing process [34, 35]. Tissue perfusion and oxygenation are also key components in wound healing [33, 36, 37]. Severe arterial insufficiency should be corrected prior to definitive treatment. Absent pulses should be investigated with Doppler studies or angiography, and angioplasty performed if this is likely to improve compromised arterial perfusion. Furthermore, any reversible cardiovascular or pulmonary condition or fluid imbalance that may interfere with adequate perfusion and oxygenation should be addressed prior to surgery [33, 36].

Endocrine disorders, such as diabetes mellitus, may also compromise surgical outcome [33, 38, 39]. Surgery induces a stress on the body resulting in the release of catecholamines, cortisol and glucagon, thereby causing surgery-induced hyperglycemia [33, 40]. To avoid intraoperative extremes regarding hypoglycemia and hyperglycemia, patients with diabetes should be examined by a dietitian and endocrinologist prior to surgery if they are not already in follow-up. If necessary, their nutritional status and insulin regimen should be corrected preoperatively [33]. Hemoglobin A1c (HbA1c) is a parameter used to determine the level of glycemic control in diabetes mellitus [38, 39]. The risk of infection in patients with diabetes mellitus increases as the perioperative HbA1c increases [38]. In patients without diabetes, hyperglycemia is also associated with poor clinical outcomes [41]. One should consider that up to one-third of intraoperative hyperglycemia cases occur in patients without diabetes [39, 40] and that, more specifically, for orthopaedic trauma patients admitted to the ICU, studies demonstrated that stress-induced hyperglycemia has a significant independent association with infection [42–46]. Therefore, glucose levels should be monitored intraoperatively in patients with and without diabetes and should be kept between 140 mg/dL (7.8 mmol/L) and 180 mg/dL (10 mmol/L) [40, 47, 48].

Malnutrition implies an imbalance of nutrients and calories required to sustain good health and development. It includes undernutrition, micronutrient deficiencies and obesity [49]. Obesity (body mass index ≥ 30 kg/m^2^), entails a risk for postoperative complications such as infection, hematoma, wound dehiscence, and, in case of soft tissue repair, flap necrosis [50, 51]. However, as surgery increases the patient’s metabolic demands, drastic weight loss prior to surgery may not be advisable [33]. A recent systematic review by Ernst et al. [52] suggests that patients who are malnourished at the time of trauma have more complicated hospital stays and increased complication rates with delayed or problematic wound healing. Patients with advanced age, recent significant weight loss and a lack of nutritional support are at greater risk of nutritional deficiency [33, 53]. Serum protein markers and weight assessment are conventional methods to assess nutritional status [33, 54]. If the patient appears nutritionally at risk, oral nutritional supplementation can be considered prior to surgery, in consultation with professionals. If the malnourishment is severe, the combination of oral nutritional supplementation with (par)enteral nutritional support should be considered prior to surgery. In such cases, if the patient’s clinical status allows it, it is advisable to delay surgery until the nutritional status is under control [53, 54].

Fracture-related infection is often connected to impaired fracture healing. A strategy that is sometimes suggested to improve bone healing is vitamin D supplementation. Vitamin D is an important factor in bone health, muscle function and the immune system [55–57]. Unfortunately, hypovitaminosis D is common and is associated with low intake, inadequate sun exposure and intestinal malabsorption. Bogunovic et al. [58] retrospectively reviewed 723 patients undergoing orthopaedic surgery. In this study, 43% of patients had insufficient serum vitamin D levels of which 40% had vitamin D deficiency. The authors concluded that, given the importance of vitamin D in musculoskeletal health, such low levels may negatively impact patient outcomes [58]. Brinker et al. performed a study on trauma patients with unexplained non-unions. Although no causative link between the presence of endocrine or metabolic abnormalities and the development of non-union could be found, there was evidence that underlying metabolic or endocrine disorders (such as vitamin D deficiency or thyroid-related problems) are common findings in unexplained non-unions [59]. The fact that endocrine disturbances seem to occur more often in this population might suggest that, in cases where the reason for non-union is unclear, referral of the patient to an endocrinologist can be considered, followed by possible supplementation and/or treatment of an underlying disorder. With this in mind, standard vitamin D supplementation for acute fracture patients could be considered. Indeed, it is known that supplementation can safely increase vitamin D serum levels and some studies also report increased bone mineral density. However, these studies focus on postmenopausal women who received vitamin D and calcium supplements over several weeks [60]. A single early high-dose vitamin D supplementation, as assessed in a recent prospective randomized controlled trial, does not seem to affect the fracture union rate [61]. In general, the evidence regarding the effect of standard vitamin D supplementation for all fracture patients—or supplementation with any other vitamin—on fracture healing and overall outcome remains scarce [60]. Therefore, it is only possible to recommend supplementation in consultation with qualified members of the MDT (e.g. endocrinologist, nutritionists) for those patients who are considered at risk, like postmenopausal women.

Many of the above-mentioned points are applicable not only to the treatment of FRI, but to all surgical subspecialties. Overall, preoperative host optimization is not always possible in FRI patients, especially in unstable fracture cases or in situations where the patient has become septic. The host status should be continuously assessed throughout the treatment course, as displayed in Fig. 1, and, depending on the assessment, optimization should start as soon as possible.

### Shared decision making

It is important that the treatment plan is established together with the MDT and adequate information is provided to the patient. FRI patients often suffer from long-term disability due to multiple reoperations, followed by prolonged hospital stays. From a psychological point of view, it is important that patients are aware of all the available treatment options including their benefits, risks and potential outcomes. In patient-centered care it is essential that healthcare professionals as well as patients can access all available information to make an informed decision, while considering the patient’s values and the healthcare professionals’ expertise or recommendations [62]. Shared decision-making is defined as the process by which the patient (or his/her significant others) makes the healthcare choices together with one or more healthcare professionals (Fig. 1) [62].

Adequate counselling of patients entails a good knowledge of the most recent scientific evidence regarding treatment of FRI. Furthermore, it is important to schedule sufficient time to allow true patient involvement in this decision-making process.

### Surgical concepts

#### Primary aims and key concepts

One of the main features that distinguishes FRI from PJI is the presence of a fracture (i.e. instability). Fracture stability is of paramount importance not only to achieve fracture union, but also for prevention and treatment of infection [63]. The classical experimental studies of Rittmann and Perren [64], showed the positive influence of stability on infection in fracture care. They stated that the advantage of the stabilizing effect of implants outweighs the disadvantage of a foreign body effect. As fracture fixation devices primarily target fracture consolidation, they can be removed after the fracture has healed (in contrast to PJI), thereby removing the biofilm and resulting in a high probability of eradication of the infection and thus prevention of chronic infection/osteomyelitis. This means that complete eradication of infection may not always be the initial goal. Suppressive therapy with antibiotics has been established as an alternative in certain cases [2, 65].

Based on this, two main surgical concepts should be considered. The first concept consists of debridement, antimicrobial therapy and implant retention (DAIR). The second consists of debridement, antimicrobial therapy and implant removal—if the fracture is healed—or implant exchange (in one or multiple stages)—if the fracture is not healed. In both cases, deep tissue sampling for microbiological examination is mandatory and adequate soft tissue cover must be achieved.

In every case, special attention should be paid to stability of the fracture in order to obtain union and treat the infection [63, 66]. Whether the implant should be removed or not, depends on several factors. The implant should, for example, be removed or exchanged (for new internal fixation or converted to external fixation) in cases where the implant and fracture are unstable, reduction is not acceptable or in severely compromised cases with poor host physiology. In isolated cases in which healing cannot be achieved due to a compromised host or due to an extensive infection, amputation or a nonsurgical approach, with or without (lifelong) antibiotic suppression may be the only treatment alternative (e.g. in elderly patients with poor host physiology) [2]. The primary aims of surgical treatment of FRI [2] are listed in Table 2.

**Table 2:** Primary aims for the surgical treatment of FRI [2]

1. Fracture consolidation 2. Eradication of infection as the final outcome (in certain cases, initial suppression of infection until fracture consolidation is achieved) 3. Healing of the soft-tissue envelope 4. Restoration of function 5. Prevention of chronic infection/osteomyelitis	

#### Tissue sampling

Confirmation of infection is achieved by culture of micro-organisms from deep tissue samples, metal implants or histopathological evaluation of deep tissue [4]. All cases of FRI require tissue sampling. Samples should be obtained early in the surgical procedure, before contamination occurs. Preferably 5 deep tissue samples should be taken with separate instruments, from sites around the fracture and adjacent to any implants [4]. The combination of microbiology and histopathology has been shown to improve the accuracy of diagnosis and allow more specific antimicrobial therapy [66–69].

#### Debridement and irrigation

Debridement remains an important surgical tool in the treatment of FRI. In general, debridement should include the excision of necrotic (i.e. non-bleeding) bone or tissue, excision of poorly perfused tissue (it will not contribute to wound healing and antibiotic delivery) and removal of all non-essential foreign bodies (e.g. broken screws, sutures) [2].

A question that sometimes leads to debate is the extend of debridement (i.e. bone resection). Although ‘oncologic resection’ of infected bone has been proposed [2], a more judicious approach is recommended with excision of bone until uniform punctate bleeding is encountered [70]. This is based on the hypothesis that infected bleeding bone is viable and can heal with antibiotic therapy (i.e. systemic and local).

Irrigation aims at decreasing the bacterial load and removal of loose debris. Most of the clinical research within this field comes from open fracture studies [71, 72]. It should be performed using normal saline at low pressure to avoid bacterial seeding in soft tissue and bone [73]. The use of additives is currently not advised as they may add to cell toxicity [71, 74]. A sufficient amount (i.e. depending on the anatomic location) of irrigation fluid should be used in order to thoroughly clean the surgical field and to lower the bacterial load after debridement.

#### Local antimicrobial therapy and dead space management

Depending on the amount of non-viable bone, adequate bone resection may create a tissue defect that must be managed. Ideally, this defect should be filled with healthy living tissue which will allow ingress of neovascularisation, delivery of systemic antimicrobials and host immune cells. The application of local antimicrobials is a powerful adjunct in the treatment of FRI and should be considered, especially in cases with a remaining bony defect or ‘dead space’ [75]. Local antibiotics may be particularly effective in chronic/late onset infections, where the perfusion of systemic antibiotic is greatly reduced due to chronic tissue scarring. A recent systematic review [76] of contaminated open fracture wounds showed a considerable risk reduction if additional local antibiotics were applied. Although PMMA was the most studied material, the prophylactic effect of absorbable carriers for local antibiotic delivery showed also promising results [76].

Indications for the use of specific types of antimicrobials, application techniques, dosages, elution properties and pharmacokinetics are poorly defined in the clinical setting leading to arbitrary variation in practice which is becoming a significant issue in orthopaedic trauma surgery [77, 78].

A detailed overview of the available local antimicrobials, including recommendations on their indications and doses, is presented in an affiliated article of the FRI consensus group [79].

#### Bone defect treatment

Segmental bone defects of long bones are challenging, especially when associated with infection [80]. A systematic review concluded that the optimal treatment depends on the surgeon’s expertise and the unique situation of the patient (e.g., infected defect size, defect location, soft-tissue envelope, medical comorbidities, patient compliance) [80]. A more recent systematic review on critical-sized bone defects in patients suffering from an FRI confirmed this conclusion [81].

#### Soft tissue management

Meticulous debridement of all non-viable tissue may result not only in bone defects, but also in major overlying soft tissue loss. It is important that orthopaedic trauma surgeons do not limit bone or soft tissue excision due to concerns about their ability to reconstruct the soft tissue envelope. Prior discussion with a plastic surgeon will allow the excision to be performed without concern about skin closure.

Data on the optimal timing of soft tissue coverage in FRI patients is limited. In cases where the soft tissue is severely compromised, a two-stage procedure may be necessary. However, if possible, a one-stage procedure can be considered and is often possible in chronic/late onset infections [82]. Single-stage treatment requires considerable logistical organization of teams. Development of good multidisciplinary collaboration is important. In any case, soft tissue coverage over infected fractures should be provided as soon as practically possible.

The choice of soft tissue reconstruction is based on many parameters. Local muscle flaps are useful in the proximal tibia and distal femur but the lower third of the tibia will require free tissue transfer. Although a recent study did indicate that a muscle flap had a lower non-union rate than a fasciocutaneous flap in open fracture cases [83], there is little evidence to recommend one specific flap type over another in FRI cases [84–88].

Finally, in the specific scenarios where multiple procedures are planned, the use of negative pressure wound therapy (NPWT) should only be used as a temporary bridge to definite soft tissue coverage. It should not be used for more than approximately 1 week and cannot serve as an alternative to definitive soft tissue reconstruction in FRI. Prolonged NPWT may lead to colonization with resistant organisms and possibly increased infection rates [89].

### Antimicrobial therapy

In most cases, antimicrobial therapy can be delayed until after deep tissue sampling. Immediate antibiotic therapy is only given to patients with sepsis and after obtaining blood for culture.

Empiric IV antimicrobial therapy should be started as soon as tissue samples are taken and maintained until definitive culture results are available. The choice of empiric therapy depends on the local epidemiology of antibiotic resistance rates, antibiotic formularies and the risk factors of each individual patient (i.e. previous antibiotics, co-morbidities, allergies, previous hospitalisations, previous debridements at the same site, previously recovered pathogens). Initially, empiric therapy should be broad spectrum, including a lipo/glycopeptide and an agent against gram-negative bacilli. Thereafter, it should be adapted according to culturing results as soon as possible. In every case, as soon as the culture results become available, antibiotic therapy should be adjusted accordingly (Fig. 1).

Duration of antimicrobial therapy is controversial and not well investigated. Overall, regimes of 6–12 weeks are common and should be decided with advice from the MDT. A recent randomized controlled trial showed that patients treated with up to 7 days of IV antibiotics followed by oral therapy had the same outcome as those with prolonged IV therapy (usually 6–12 weeks) [90].

When a segmental excision has been performed and all dead and infected tissue removed, it may be sufficient to give 2 weeks of antimicrobial therapy to eradicate the residual contamination in the soft tissues. However, in case of suppressive treatment, therapy should be continued until fracture union.

In cases with a low suspicion of infection, it is safe to give empiric antibiotic therapy until culture results and histopathology is available. Therapy should be stopped as soon as infection is ruled out.

As presented in Fig. 1, all FRI patients should have a baseline blood analysis available, especially patients who will receive IV antimicrobial therapy, including baseline inflammatory markers [e.g. C-reactive protein (CRP)], full blood count, electrolytes and liver- and renal function tests. Depending on the type of antibiotic and local preferences, some specific blood parameters (e.g. liver function tests for rifampicin, electrolyte levels and full blood count for fluoroquinolones, etc.) should be monitored at least twice weekly, as common side effects of high-dose IV antibiotics include bone marrow suppression, hepatitis and nephrotoxicity. Sometimes, it is not possible to use oral antibiotic therapy, mainly due to intolerance or microbial resistance. In these cases, an out-patient parenteral antibiotic therapy (OPAT) service reduces costs and can support patients at home, with monitoring of therapy.

A more extended review with recommendations regarding antimicrobial therapy in FRI patients is presented in an affiliated article of the FRI consensus group [91].

### Rehabilitation and follow-up

Early individualized functional rehabilitation is a critical aspect for every orthopaedic trauma patient and particularly when infection arises. Therefore, physiotherapists and physical medicine and rehabilitation physicians play a key role (Fig. 1). As FRI often entails a prolonged treatment pathway, psychological support should also be available. In these cases, specialist nurses can play a key role.

Patient optimization principles, as discussed in “Host optimization”, should also be implemented in the postoperative and follow-up period. A follow-up of minimum 12 months after cessation of (surgical and antibiotic) therapy is required. The follow-up frequency depends on local preferences and policies and on the extent of the infection. Follow-up outpatient visits generally consist of a wound inspection, radiological evaluation of the fracture and monitoring for complications or recurrence of infection. In case of deterioration of the patient’s health status (e.g. sepsis), CRP levels can help the decision-making process, although physicians should be aware that the evidence for the use of serum markers as diagnostic and follow-up parameters is scarce [92].

Regarding the standard removal of implants after consolidation/healing of a sustained FRI, currently insufficient evidence exists to recommend this at a routine basis.

### Standardized patient outcome measures

#### Clinical versus patient-reported outcomes

Standardized outcome measures for FRI patients are currently lacking. Such outcomes include clinical outcomes, functional outcomes, general health-related outcomes, and satisfaction with the process of care. Clinical outcomes (such as range of motion, radiographic union, implant loosening, and ongoing or recurrent infection) have been the focus of clinical research in orthopaedic surgery [93, 94]. However, good clinical outcomes do not necessarily indicate a good return to function. Functional outcomes primarily involve the patient at the most complete level, not as a joint or condition but as an individual in society [95–97]. Health-related quality of life involves patients’ perception of how they are functioning as affected by their overall health. Covered areas of individual function include mental health, social function, role function (i.e., worker, spouse, parent), physical function, and activities of daily living [98, 99]. Patient-reported outcomes will become more important for reporting quality metrics, for insurance companies, and for the internal evaluation of patient outcomes, as well to be able to compare different anatomic sites. No official recommendation on this topic has been made by international organizations for trauma patients and further research on this topic is warranted.

#### CAT instruments

Computer adaptive testing (CAT) greatly decreases the patient burden to acquire data. The well-established Patient Reported Outcomes (PROs) used in trauma that do not involve CATs are the SF-36, the Sickness Impact Profile (SIP), the Western Ontario and McMaster Universities Arthritis Index (WOMAC), the Nottingham Health Profile, Quality of Well Being (QWB), Musculoskeletal Functional Assessment (MFA) and the EQ-5D [100]. Furthermore, within musculoskeletal health, multiple scores have been developed to evaluate individual body parts or joints. These global and injury-specific instruments have been validated and used as qualitative assessments of patients’ overall health and function. They have the advantage of being consistent across patients and being repeatable measures. However, they have the disadvantage of having to repeat all questions to each respondent at each evaluation, which leads to patient burden, subject fatigue, and subsequent loss of compliance or follow-up [101]. In contrast, CAT provides the opportunity to collect equally accurate assessments of patient function while asking far fewer questions, which should enable the collection of data from more patients, more often, with less patient fatigue in the data collection process.

Computer adaptive testing utilizes “item response theory” and is considered a dynamic assessment. This approach enables patients to answer questionnaires with far fewer questions with near equal specificity of a patient’s level of function and sensitivity to changes in function as the full version of scientifically developed questionnaires.

The most widely employed patient-reported outcome (PRO) CAT is the ‘patient-reported outcomes measurement information system’ (PROMIS) instrument, the development of which was funded and backed by the NIH. The PROMIS scores focus on domain-specific assessment (physical function, mental health, etc.), rather than the injury-specific focus of some legacy instruments. By providing equal or improved accuracy of assessment with substantially less patient burden, PROMIS is a research instrument for assessment of lower extremity trauma patients with or without an FRI. Subsequent evaluation of PROMIS for the upper extremity has also been encouraging [102, 103]. Other CATs exist, but are less widely employed to date and warrant further investigation.

Due to the significant decrease in patient burden and equal ability to thoroughly evaluate patients function and changes in function, computer adaptive testing is likely to be common in future outcome assessments, also for FRI patients. At this time, it appears that NIH PROMIS is the most likely future assessment tool for most orthopaedic trauma research.

## Conclusion and recommendations

FRI remains a challenging complication. To improve overall outcome, we should aim for standardized recommendations for diagnosis and treatment. This review focuses on delivering these recommendations (Table 3), in combination with an optimal treatment pathway for FRI patients (Fig. 1) based on up-to-date scientific evidence and expert opinion.

**Table 3:** Key recommendations

A well-established diagnosis is the first step in the treatment process of FRI patients. The presence of confirmatory signs should prompt treatment for FRI. Suggestive signs should motivate the medical team to further investigate the probability of an FRI A multidisciplinary approach is a key aspect in FRI treatment and should be implemented. The exact composition of the MDT will depend on the patient’s needs and local preferences It is recommended to refer complex cases to specialized centers where an MDT is available and physicians are experienced with the treatment of FRI The patient’s health status should be optimized. Optimization strategies should be started in consultation with the MDT and preferably preoperatively, if the clinical status allows it Patients who are nutritionally at risk for malnutrition should be considered for screening and, depending on the severity, a multidisciplinary approach (e.g. endocrinologists, nutritionists, geriatrics) for the optimization of this status should be implemented Fracture stability is of key importance with respect to the surgical treatment of FRI Thorough debridement is essential as well as adequate management of the dead space that may be created Low-pressure irrigation should be performed with a sufficient amount of normal saline in order to thoroughly clean the surgical field and to lower the bacterial load The application of local antimicrobials should be strongly considered NPWT should only be used as a short bridge to definite soft tissue coverage In case of FRI, start empiric broad-spectrum antibiotic therapy after tissue sampling A minimum follow-up of 12 months after cessation of (surgical and antibiotic) therapy is recommended, with the follow-up frequency depending on local policies and preferences Standardized patient outcome measures for FRI are currently not available. PROMIS seems to be the preferred tool to assess the patients’ short and long-term outcome	

*FRI* fracture-related infection, *MDT* multidisciplinary team, *NPWT* negative-pressure wound therapy, *PROMIS* patient-reported outcomes measurement information system

Starting from the first patient contact, a full diagnostic work-up should be performed, considering the recently published diagnostic criteria [3, 4]. The presence of suggestive signs should encourage the MDT to further investigate the probability of an FRI and to look for confirmative signs. The presence of confirmative signs should prompt treatment, based on host type and a multidisciplinary approach. If the patient is otherwise healthy the treatment plan can be started immediately. In the case of compromised hosts—based on the ASA score and assessment of the local health status—patient optimization should be initiated as soon as feasible, depending on the clinical status (e.g. sepsis, severe bony instability) of the patient.

Surgical treatment entails multiple key aspects (e.g. sampling, debridement, local antimicrobial therapy, soft tissue management, bone defect reconstruction). A judicious well-planned debridement remains one of the cornerstones in the treatment of FRI. The surgeon should give special attention to obtaining uncontaminated deep tissue samples for microbiology and histopathology. Immediately after tissue sampling, empiric broad-spectrum antibiotics should be started in case an FRI is suspected. Based on the result of tissue cultures and the corresponding antibiogram(s), antibiotic therapy needs to be adapted. OPAT can be considered in cases where longstanding IV antibiotic therapy is necessary. Regular follow-up is needed to monitor therapy, identify complications early and maximize functional outcome. Finally, follow-up of a minimum 12 months after cessation of (surgical and antibiotic) therapy is recommended.

Clinical outcomes of fracture union and absence of infection recurrence are important, but standardized patient-reported outcome measures are also critical.
